# Systems-based approaches for investigation of inter-tissue communication[S]

**DOI:** 10.1194/jlr.S090316

**Published:** 2019-01-07

**Authors:** Marcus M. Seldin, Aldons J. Lusis

**Affiliations:** Departments of Medicine,* University of California, Los Angeles, Los Angeles, CA 90095; Human Genetics† University of California, Los Angeles, Los Angeles, CA 90095; Microbiology, Immunology, and Molecular Genetics§ University of California, Los Angeles, Los Angeles, CA 90095

**Keywords:** cytokines, diabetes, genetics, hormones, receptors/hormone

## Abstract

Secreted proteins serve as crucial mediators of many physiology processes, and beginning with the discovery of insulin, studies have revealed numerous context-specific regulatory networks across various cell types. Here, we review “omics” approaches to deconvolute the complex milieu of proteins that are released from the cell. We emphasize a novel “systems genetics” approach our laboratory has developed to investigate mechanisms of tissue-tissue communication using population-based datasets. Finally, we highlight potential future directions for these studies, discuss several caveats, and propose new ways to investigate modes of endocrine communication.

## Graphical Abstract

**Figure:**
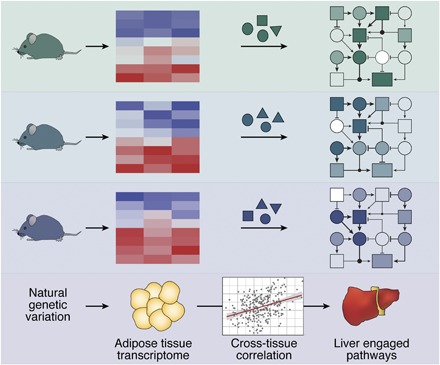

## SYSTEMS GENETICS

Systems genetics, an approach that involves the integration of clinical/physiological and omics level data in natural populations, is proving particularly useful for the analysis of complex traits (1). For example, quantification of global mRNA levels for a particular tissue in a population can be used to identify common genetic variants that control transcription, termed expression quantitative trait loci (eQTLs). These can be related to clinical traits by co-mapping, correlation or statistical modeling to help identify candidate disease mechanisms. In this review, we discuss the application of systems genetics approaches to identify novel endocrine factors mediating tissue-tissue cross-talk.

## ABUNDANCE OF SECRETED PROTEINS IN MAMMALS

Secreted proteins constitute a significant fraction (roughly 15–20%) of the coding genome (2). These serve a variety of functions, including the relay of signals to neighboring cells, a process conserved from bacteria to humans. Since the discovery of insulin over a century ago, many elegant studies have related circulating proteins to regulatory and homeostatic functions, and these proteins have been found to be involved in many common human diseases. Research on molecules, such as leptin, adiponectin, ghrelin, and fibroblast growth factor family members, has driven our understanding of endocrine metabolism and guided therapeutic development for diseases such as obesity and type 2 diabetes. At present, hundreds of additional secreted proteins have been described to regulate diverse cellular processes, elucidated largely through gain- or loss-of-function studies. As a result, various clinical trials have shown promise in modulating endocrine factors’ levels in circulation through antibody-based inhibition or direct administration of recombinant proteins. Understanding of the functions of the vast majority of secreted proteins, however, is still lacking, presenting an exciting avenue for scientific discovery and therapeutic development.

## ASSAYS AND METHODS TO INTERROGATE CELLULAR SECRETOME

The past decade has seen an immense increase in the generation of large omics datasets aimed at generating a more global picture of cellular states. Specifically, advances in efficiency, cost reduction, and analytical approaches to interpret transcriptomic (expression arrays or RNA-sequencing) and/or proteomic (multiplexed antibody/aptamer or mass spectrometry-based quantification) have further helped to guide our understanding of the cellular “secretome”. Gene expression or quantitative protein datasets have been filtered for either the predicted or annotated presence of secretion signals to gain insight into context-specific regulation of the secretome at the transcript level (3, 4). Likewise, proteomics can be performed directly on culture media (5–7) or plasma (8–10). Several recent studies have identified novel genetic mechanisms of the circulating proteome that show enrichment in disease (11, 12). Some relative advantages and disadvantages of each strategy have been described in greater detail (13–15). A recent study performed cytokine profiling in blood cells under a variety of pathologic stimuli from 500 healthy individuals. By pairing these observations with genetic data, the authors provided a snapshot of genetic regulation of the secretome under normal or perturbed conditions (16). These generalized approaches have substantially contributed to our understanding of cellular regulation of secretory pathways and revolutionized biomarker discovery. Given that these observations are mostly correlative in nature, it can be quite difficult to infer the physiologic functions of the secreted molecules identified and determine whether they are causal or reactive with respect to a physiological or disease process.

## DECONVOLUTION OF ENDOCRINE INTERACTIONS USING NATURAL VARIATION

Combined analyses of omics data across populations have been instrumental for inference of pathways regulating diverse cellular and tissue functions. One such example includes the STARNET (Stockholm-Tartu Atherosclerosis Reverse Network Engineering Task) population, where genotyping was combined with RNA-sequencing from several tissues to guide disease inference (17). This initial study also noted strong enrichment of human genome-wide association study disease loci in networks that persisted across tissues, suggesting an important role for signaling between multiple tissues in disease progression. Further, the notion that a gene network model across tissues could explain genetic differences between susceptibility to pancreatic islet dysfunction was put forth by comparing two strains in mice (18). In 2016, Long et al. (19) constructed a coexpression network of heart and blood transcripts from the GTeX (Genotype-Tissue Expression Project) dataset, noting that a secreted protein, dipeptidyl peptidase 4, could act as a link mediating signaling between the tissues (19). These studies and several others highlight the potential of comparing gene expression across tissues from genetically diverse individuals.

**Fig. 1.**
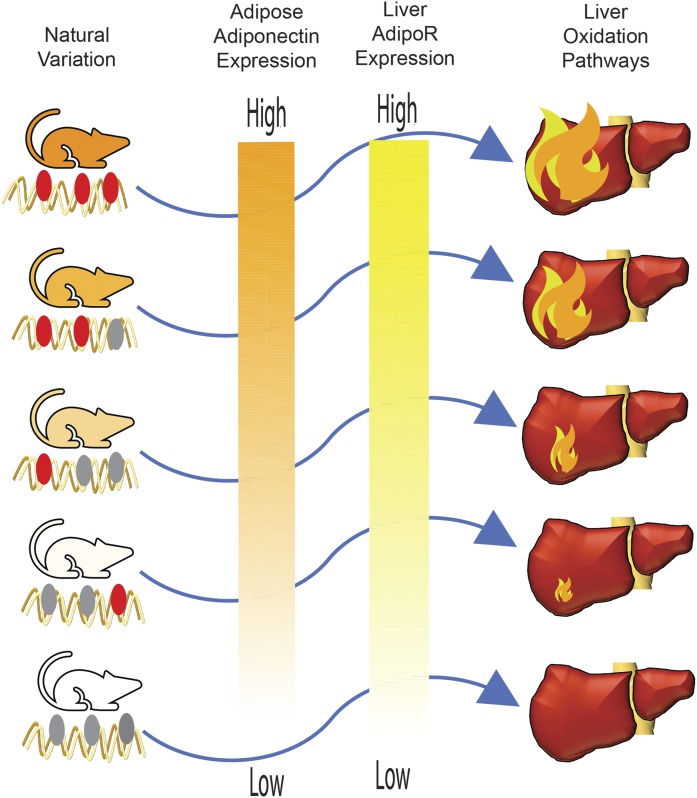
Intuition for QENIE. Illustration of the intui­tion for QENIE approach from adipose tissue to liver. Natural variation drives different levels of adipose tissue expression of an adiponectin (left). Given that adiponectin engages hepatic oxidation pathways through its receptor, the abundance of AdipoRs and consequent oxidative pathways (right) will show similar quantitative levels to that of adiponectin in fat.

We recently developed a framework that utilizes natural variation and multi-tissue datasets to identify modes of endocrine communication, termed Quantitative Endocrine Network Interaction Estimation (QENIE) (20). The basic concept is simply to search for genes that strongly correlate with transcriptome measures across tissues within a population. For our study, we used the hybrid mouse diversity panel (HMDP), a collection of about 100 “classical” as well as recombinant inbred strains of mice. Using global transcriptomic data from several tissues, we found that correlation structure between tissues predicted many known interactions, such as adiponectin acting from adipose tissue to liver. The intuition underling this prioritization is that the hepatic pathways and functions engaged by adiponectin would be apparent through correlations with the gene in adipose tissue (**Fig. 1**). For genes exhibiting such correlation structure, we hypothesized that we could use pathway enrichment analysis and integration with available clinical trait data to generate testable hypotheses as to function. Using this approach, we identified and characterized several novel genes that mediate signaling and affect functions across tissues. For example, we showed that adipose-derived lipocalin-5 is an enhancer of skeletal muscle respiration, and that liver-derived Notum is a promoter of adipose tissue thermogenesis. We also provided evidence that inter-alpha-trypsin inhibitor heavy chain family member 5 suppresses heart starvation response, that SPARC-related modular calcium binding 1 regulates cardiac cell proliferation, and that pro-platelet basic protein in enhances vascular inflammation. This approach is generalizable in that it can be easily applied to mouse or human or other populations that have been subjected to appropriate transcriptomic or proteomic analyses.

## FUTURE APPROACHES TO INVESTIGATE TISSUE-TISSUE COMMUNICATION

**Fig. 2.**
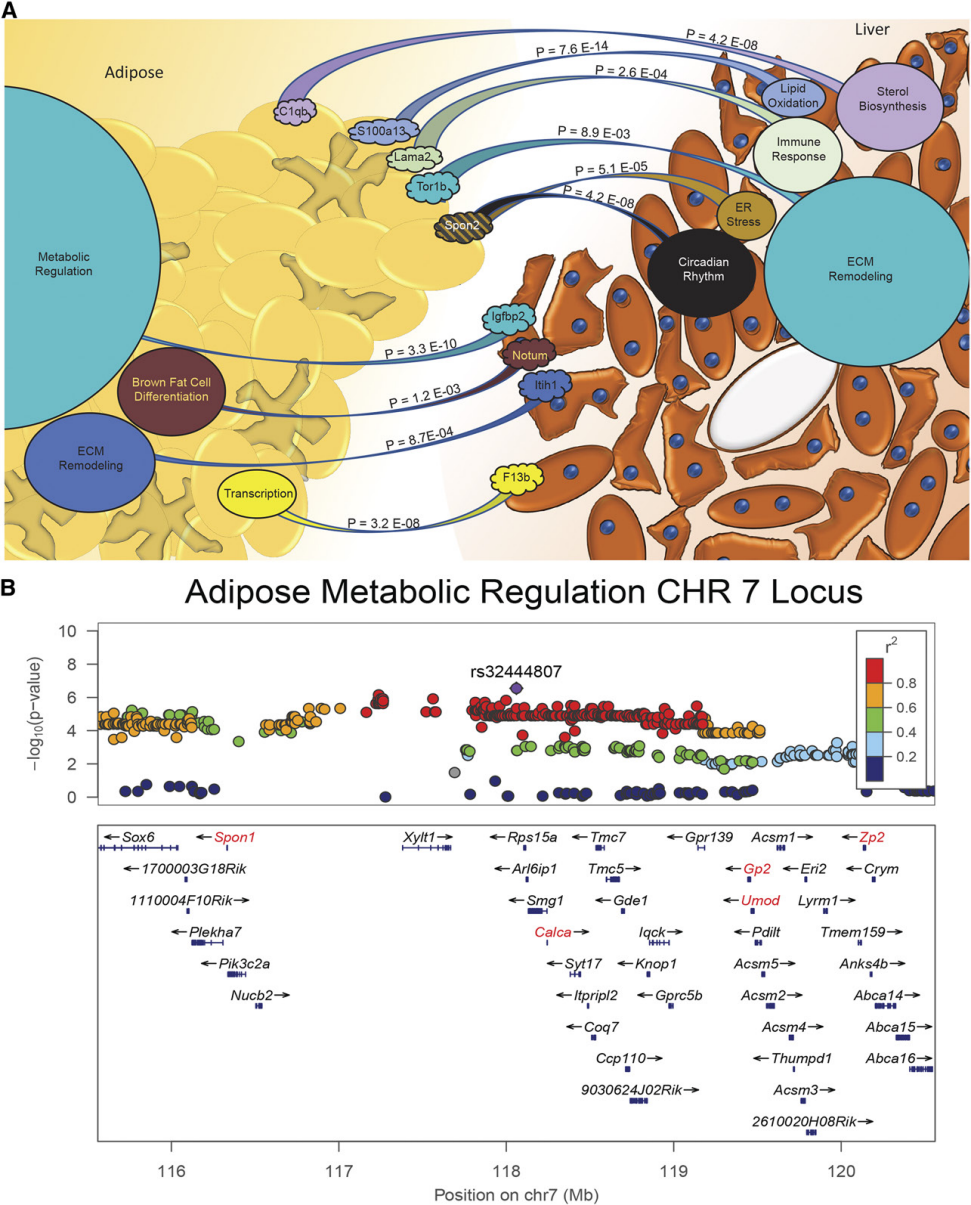
Pathway-based interactions between liver and adipose tissue. A: Each pathway was constructed from weighted gene correlated network analysis (WGCNA), where the size of the circle indicates the number of genes in each respective module. The strongest predicted genes encoding secreted proteins in the neighboring tissue for each module is shown, with the *P* value based on permutation testing. B: Locuszoom plot showing a genome-wide significant region on chromosome 7 for the adipose tissue metabolic regulation module eigengene. Factors that are secreted are highlighted in red.

Our initial framework begins by ranking interactions by global patterns of correlation, which biases predictions toward larger pathways of target tissue genes. An alternative approach would be to focus on specific pathways or correlated modules of genes in a given target tissue, then rank origin tissue secreted factors based exclusively on these genes. This is illustrated in **Fig. 2A** between liver and adipose tissue, where genes predicted to target individual modules are indicated. Here, an unbiased approach to network construction, weighted gene correlated network analysis (WGCNA), elucidated several different modules of correlated genes. These individual modules were enriched for very different pathways, which in turn were correlated with different secreted proteins in the reciprocal tissue. It is important to note that regardless of how the interactions are constructed, experimental validation is required, as it is impossible to eliminate all confounding factors. Along these lines, a different approach could utilize regulatory genetic variation to drive correlations. For example, focusing on a *cis*-eQTL for a given secreted protein, which also maps in *trans* to a target tissue pathway, could provide evidence of causal inference. However, one caveat with this approach would be the exclusion of *trans*-regulated genes coding for secreted proteins. Given that, generally, *cis*-regulation tends to explain a small fraction of variation in gene expression as well as trait association (21–23) and that several well-described endocrine factors, such as adiponectin, leptin, resistin, and angiopoietin-like 4, are not regulated in *cis* in adipose tissue within the HMDP, we chose to focus on a correlation-based approach.

Perhaps the most obvious potential future application of QENIE or similar approaches is to integrate quantitative proteomics data. This can be accomplished by either *1*) using tissue-specific proteomics data to make similar cross-tissue predictions or *2*) pairing either tissue gene expression and/or proteomics correlations with quantitative plasma protein data to refine predictions. Here, one could simply correlate pathways in tissues with adipose and/or plasma levels of a secreted factor. For example, the heterotrimeric structure of secreted adiponectin has been shown to play an integral role for its function, which could be measured in the HMDP and presumably correlate even more strongly with liver oxidative gene expression (24–26). Given that levels of protein in a specific tissue or plasma are much closer to the mode of action, these analyses are potentially more powerful than gene expression. Similar to reliance on correlation with transcript levels, integration of quantitative trait loci regulating proteins would further aid causal inference. This would be particularly valuable in instances where endocrine regulation of a specific pathway is being driven by posttranscriptional control of a circulating factor. Several notable studies have described distinct patterns of *cis*-regulated proteins, which cannot be detected at the transcript level (27). One constraint of using proteomics is the limitation of depth, as RNA-sequencing tends to reliably detect around 4- to 6-fold more coding genes when compared with mass-spectrometry proteomics.

As mentioned above, one important constraint in the current QENIE pipeline is the exclusive reliance on correlation to predict interactions. While we feel that this enables the most potential for discovery of endocrine circuits, this approach is subject to spurious correlations and therefore, requires substantially more experimental validation than other predicted (e.g., overlapping *cis*-eQTLs with modules in other tissues). Given that many correlations are reactive in nature, many cross-tissue predictions follow the same pattern. For example, if both the expression of a secreted factor in one tissue and the global pathway in another are regulated by insulin, correlation would show a direct relationship, which may be spurious. One could regress these correlations on plasma insulin to tease out these effects; but given the complexity of these processes, it is quite difficult to pinpoint potential sources of spurious relationships. Consistent with these considerations, we have observed an approximate 15–25% “validation rate” of such interactions, depending on dataset used. For this reason and others, it is crucial to follow predictions with experimental validation.

An alternative to identification of endocrine regulation through co-mapping of *cis*-eQTLs or *cis*-pQTLs with pathways in neighboring tissues, which would likely improve causal inference from QENIE, would be to simply evaluate genes near a locus for the pathway of interest. Here, the approach would be to map modules of gene expression and look for potential secreted factors under the locus. To demonstrate the utility of such approaches, genome-wide association was performed on the eigengene for the adipose tissue metabolic regulation module, where several endocrine factors surrounding a significant quantitative trait locus are highlighted in red (**Fig. 2B**). The appeal here would be that there is little reliance on whether a *cis* variant is acting at the level of gene, protein, or secretion; however, without integration of additional data uncovering which protein is driving the module and what tissue is the primary contributor to the observed effect would be difficult.

Additionally, one could integrate this approach with metabolomics datasets. In our initial study, we compared the average level of correlation across tissues for genes coding for secreted proteins versus all other genes, where most tissue-tissue pairs showed stronger correlation enrichment for secreted proteins. We noted several exceptions to this observation. For example, nonsecreted genes from skeletal muscle correlated more strongly than those of secreted proteins across the adipose tissue transcriptome. One possible explanation for this observation is that these genes are involved in the production of a metabolite that relays a signal between the two tissues. Previous studies have described a strong genetic influence of the plasma metabolome (28–31) and, given that many of these molecules are known to relay signals between tissues, one could expect to uncover some new functions by integrating the data in a similar manner.

An area of research that has gained considerable recent attention is the investigation of circulating microRNAs as signaling mechanisms between tissues. These microRNAs can be found either packaged in lipoprotein particles (32) or exosomes (33), or bound directly by protein complexes (34), and can impact a variety of tissues. These microRNAs could also serve as the basis for analyses of communication across tissues, as opposed to proteins, which are secreted.

Beyond modification of the pipeline or application to other datasets, there are some intriguing future directions for some of the endocrine factors we screened and identified. For example, identification of the upstream physiologic parameters that regulate expression and secretion of the proteins will be crucial to uncovering their conserved roles. Whether genes such as lipocalin-5 and Notum are regulated by fasting/feeding, exercise, or cold exposure could guide future studies. Further, knowing the receptors by which these proteins act would be highly informative as to their functional and tissue-specific impacts. While the receptor by which lipocalin-5 signals is not known, Notum has been shown to be a potent inhibitor of Wnt signaling (35). From these known observations, one can begin to ask how Notum links Wnt signaling inhibition to adipose tissue thermogenesis.

In conclusion, systems genetics approaches provide a powerful complement to traditional methods for endocrine discovery. Much remains to be learned because the functional outcomes of hundreds of secreted proteins remain unknown. A major challenge is the understanding of how these novel factors function in homeostasis and contribute to disease.

## Supplementary Material

Supplemental Data
