# Supplementary material for: Systems-based approaches for investigation of inter-tissue communication
Source: J Lipid Res. 2019 Jan 7;60(3):450–5. doi: 10.1194/jlr.S090316 (PMC6399495; doi:10.1194/jlr.S090316)
Supplement: Supplemental Data [file supp_60_3_450__index.html]

Systems-based approaches for investigation of inter-tissue communication — Systems-based approaches for investigation of inter-tissue communication — Supplemental Data 

# Systems-based approaches for investigation of inter-tissue communication

## Supplemental Data

- Graphical Abstract (.jpg, 369 KB) - Graphical Abstract prepared by Luciana Giono
