# Supplementary figures and images for: Systems-based approaches for investigation of inter-tissue communication
Source: J Lipid Res. 2019 Jan 7;60(3):450–5. doi: 10.1194/jlr.S090316 (PMC6399495; doi:10.1194/jlr.S090316)

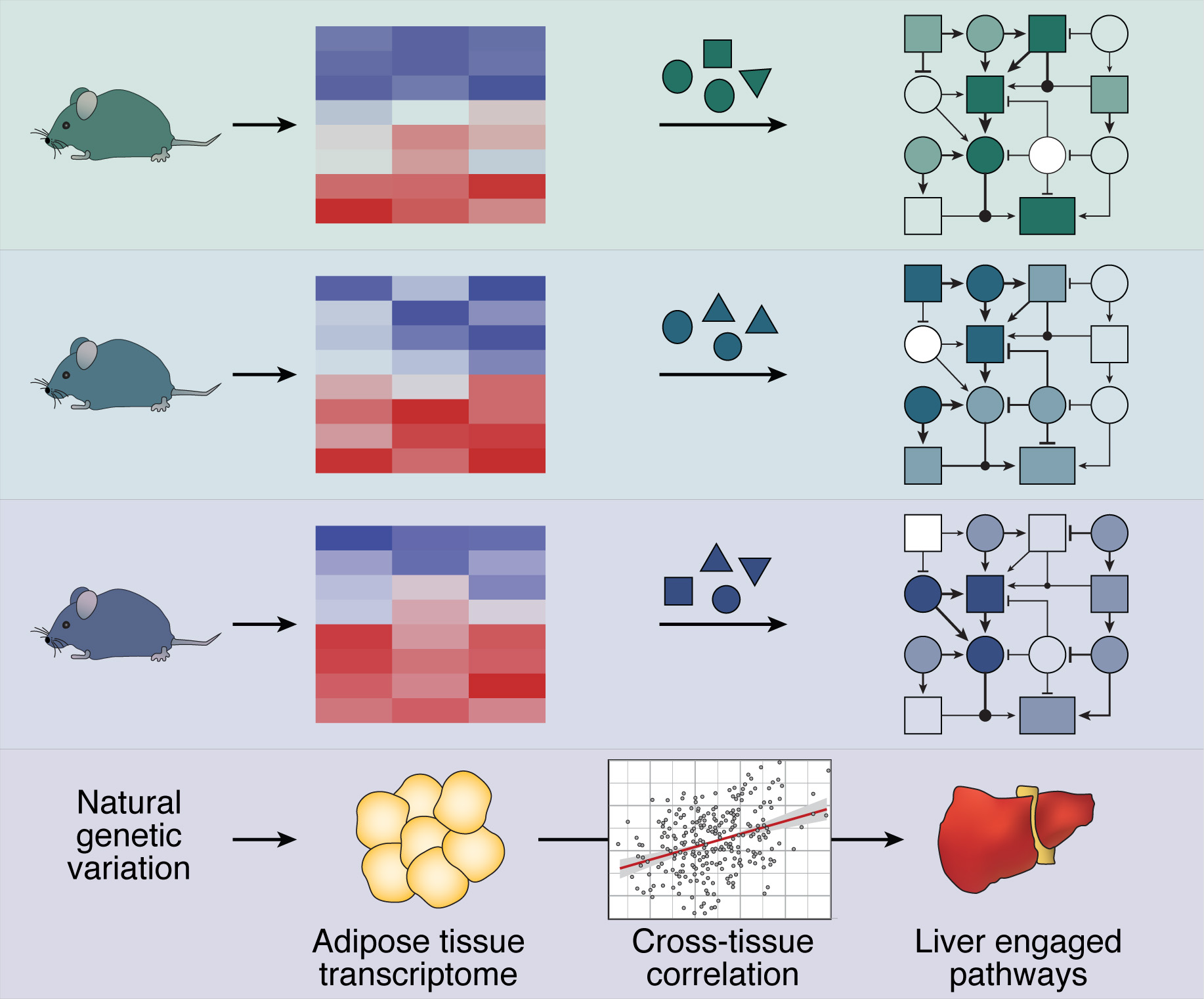

Supplement: Supplemental Data [file 10.1194_S090316_jlr.S090316-1.jpg]
